# Supplementary material for: Visual attention towards food during unplanned purchases – A pilot study using mobile eye tracking technology
Source: PLoS One. 2021 Mar 4;16(3):e0247755. doi: 10.1371/journal.pone.0247755 (PMC7932502; doi:10.1371/journal.pone.0247755)
Supplement: Attachment — Submitted filename: PONE-D-20-12685_reviewer1.pdf [file pone.0247755.s001.pdf]

Referee Report for PONE-D-20-12685

Visual attention towards food during unplanned purchases— A pilot study using mobile eye tracking technology

This papers uses a field experiment to examine the relationship between individuals' weight status, food energy density, and visual attention during unplanned purchases. Results indicate that participants with higher BMI show an attention bias towards high-calorie foods compared to low-calorie foods. The opposite effect is found for subjects with lower BMI.

There is much to like about this paper. It addresses a very important public health problem (unhealthy eating) in a novel way. It considers distributional effects across relevant groups (i.e. BMI categories), which are generally understudied. Having said that, I have major concerns which are mainly related to the lack of information on the experimental procedures and the small number of observations. Please find my concerns below.

Main Comments

1. My biggest concern relates to sample size. There are two weight classifications (high and low BMI), with less than 10 subjects in each category. This is a low number unlikely to be supported by an ex ante power calculation. I understand that obtaining a large number of participants in a real setting, particularly subjects with high BMI, can be somewhat of a challenge. But any statistical analysis based on such few subjects would, at best, be highly preliminary. I would encourage the authors to increase their sample size significantly.

2. Subjects are classified into two weight categories based on self-reported measures of height and weight. Since BMI classification is a main component of this study, relying on self-reported measures is not ideal. If the authors are to increase sample size, I suggest they look into ways to collect weight and height measures during the experiment; for example, experimenters could collect these measures at the end of the last shopping trip, right before payment.

3. I find the description of experimental procedures to be exceptionally sparse. When was the eye tracking data collected? Was the same weeks used for all subjects? (e.g. eye tracking data was collected for all subjects in weeks 2 and 4), Did subjects knew they would be using eye tracking glasses during those trips beforehand?

4. How many online questionnaires were collected by subject and when were these implemented? What information did they collect besides socio-economic characteristics? This is important because the type of questions could prime subjects and affect their subsequent purchasing behavior. For example, it would make a big difference if the self-reported weight and height measures were collected at the beginning or at the end of the experiment as asking for subjects' weight might prime them towards specific food products or product quantities.

5. Who are the participants? The authors mentioned that socio-economic characteristics were collected in the online survey (e.g. gender, age, income); however they do not provide any description of the sample population. I find it a significant omission for an experimental paper to not present a summary of the demographic profile of the sample. This is not necessarily a criticism of the experiment procedures, but rather the written summary of the procedures. I suggest the authors provide a table with a summary of the demographic profile for all the sample and by BMI category (normal weight and overweight/obese).

6. Each participant received a 50 € voucher as compensation fee at the end of the experiment. Was there another incentive throughout the experiment? If subjects knew they would be receiving the voucher for completing the study, there was no incentive for them to truthfully report their preferences during each shopping visit (i.e. while making shopping lists and purchases). The compensation should have been split into weekly payments.

7. Why was participation restricted to households with 2-5 members? Were participants shopping by themselves? This is important as the presence of a second individual might steer participants toward specific products. For example, Papoutsi et al. (2013) show that children's pestering power strongly affects parents in making unhealthier food choices. I suggest the authors use data from the online questionnaires (e.g. Who joined the trip?) to control for the number of people in the shopping trips as well as household size.

8. Subjects were asked to write a weekly shopping list and collect grocery receipts for four weeks. When were the receipts and lists collected by the experimenter? If these were collected at the end of the experiment, subjects had the opportunity to revise their shopping lists as much as they wanted. Also, there is the possibility that after 1-2 weeks subjects got an idea of the purpose of the experiment and started adjusting their behavior. I suggest the authors control for week effects.

9. The results in Table 1 and Table 2 should be split by BMI and energy density. For example, I would like to see the number of LC and HC unplanned purchases for each BMI category, same for all statistics reported in Table 2. This might cause the significant effects in Table 2 to disappear due to the small sample size, which highlights the need to collect more data.

10. Throughout the paper, there is no mentioning of the type of statistical tests used (t-tests, one or two-sided, etc.) when statistical analysis is performed. This information needs to be clarified, particularly in Tables and Figures. The authors refer to p-values in Table 2 and Figure 2– what are the statistical tests? The authors should include the relevant missing information in the table notes and figure captions so that they are self-contained.

11. The analysis is generally lacking in statistical rigor. The results are primarily based on pairwise-comparison tests and I think there are several important factors that need to be controlled for using model specifications/regressions. For example, I would strongly encourage the authors to explore whether the results vary over time (by week); are the findings consistent if one looks at early purchases (e.g. first 2 weeks) vs. later purchases (2 latest). The authors should also control for time-of-the-day effects and individual characteristics such as hunger level, gender, household size, income, eating habits.

12. Related to my previous comment, the authors are using the observations (unplanned purchases) made by the same individual in different weeks as independent observations. I suggest they

consider using models for panel data that cluster the standard errors at the individual level and control for all factors described in the previous comment. They can also test for interaction effects between weight status and energy density using these models rather than ANOVA tests (or complement both).

13. The authors compare attention bias towards the bought product and the labels. What information was provided in the shelf labels? If the only information provided was the product price, a possible explanation for the lower amount of time spent looking at the labels compared to the product could be the familiarity with this attribute. It is reasonable to think that since individuals compare product prices on a regular basis, they might not need as much time to process price information. I think the authors could make more use of the eye tracking data by creating AOIs for the product labels instead of shelf labels. They could explore whether subjects fixate longer on calorie content or health claims such as low-fat, sugar reduced, fat-free, etc. and relate this to unplanned food choices.

14. It would have been interesting to see more results related to heterogeneity of unplanned purchases/visual attention of individuals who were more/less hungry according to the scale the authors collected. There is a vast literature on how hunger might produce different effects, and this paper could have something to say about this.

Other comments

1. The relationship between visual attention and food choice across BMI categories is an important contribution that has been highly understudied. Only few studies that have examined such distributional effects (Segovia et al. 2019). This is worth stressing more in the paper.

2. The authors should be consistent in the terminology used for BMI categories, sometimes they refer to the groups as high vs. low BMI, sometimes as BMI<25 vs. BMI>25, and other times as normal weight vs. overweight/obese.

3. Are there underweight subjects in the sample? (BMI< 18.5). If so, they should not be part of the normal weight group.

4. Page 15, lines 357-358: sentence makes no sense. Please edit.

5. The limitations of the study can be discussed in the conclusions section, no need for a separate section.

References

Papoutsi, Georgia, Rodolfo Nayga, Panagiotis Lazaridis, and Andreas Drichoutis. "Nudging parental health behavior with and without children's pestering power: Fat tax, subsidy or both?." (2013).

Segovia, Michelle S., Marco A. Palma, and Rodolfo M. Nayga Jr. "The effect of food anticipation on cognitive function: An eye tracking study." *PloS one* 14, no. 10 (2019): e0223506.
