# Supplementary material for: Visual attention towards food during unplanned purchases – A pilot study using mobile eye tracking technology
Source: PLoS One. 2021 Mar 4;16(3):e0247755. doi: 10.1371/journal.pone.0247755 (PMC7932502; doi:10.1371/journal.pone.0247755)
Supplement: Attachment — Submitted filename: Rebuttal letter that responds to reviewers and editors.pdf [file pone.0247755.s006.pdf]

Dear Reviewers, dear Editor.

With the help of the reviewers and the editor, we have been able to steadily improve the publication to this day. We are aware of this development and the understanding of the reviewers and thank them for their support throughout the whole review process.

We understand that the paper in its current version still has some weaknesses that need to be improved before publication. Some misunderstandings and format issues have arisen. These include the issues raised by the reviewer, which we will address now.

1) In our last review, particularly in the response letter to the reviewers, we indicated that we controlled for the influence of the arrangement. Unfortunately, we were a little too imprecise. We did not mean that we controlled for the arrangement by randomizing the starting point as reviewer 2 points out. This would have been a very appropriate control method for testing the influence of the arrangement for an experiment. Nevertheless, our study describes much more an observational study in which we as scientists have only a very limited influence on certain variables, including the arrangement in the supermarket. We were interested in observing and evaluating spontaneous purchases in situations that were as natural as possible and not 'artificial'. A high external validity was clearly the focus of our study. The benefit of this study is therefore the recording and analysis of realistic shopping situations and behavior in a real-world setting. Therefore, we had to forgo many experimental controls such as testing the influence of the arrangement by varying the starting point. In addition, varying the starting point would also have led to people having to walk unrealistic distances, possibly even having to walk distances twice or three times to be able to buy all the products. The time of the purchase would have changed with it and as far as time and time pressure are really connected as reviewer 2 assumed, the time pressure would have changed with it as well. Thus, varying the starting point in a realistic setting does not lead to the desired controls without significant side effects. The research team had already thought about these conditions before the study and deliberately refrained from varying this factor in any way. In our opinion, this inevitably leads to more unrealistic conditions and thus contradicts our fundamental intention. Accordingly, we followed observational studies and collected such influencing variables with our data to be able to statistically control for them as part of our analyses afterwards. And that is what we mentioned when we wrote we controlled for this variable. Since the reviewer also pointed out deficiencies in the manuscript in this respect, we added an additional part in the discussion describing this issue again in more detail and justifying our approach. Furthermore, we have extended the discussion regarding time and time pressure and a possible confounding with eye movement measurements and pointed out more clearly and again possible limitations of our results. We hope we were able to satisfy the reviewers and editors sufficiently regarding this point. We hope we were able to satisfy the reviewers and editors sufficiently about this point.

2) I am personally very embarrassed by this point, especially the formatting. The way it is presented is in no way appropriate. Unfortunately, I had copied the table into the text template and assumed that the formatting would be preserved. This was not the case. In the future I will check this several times or use figures of the tables if necessary. To provide the tables at least now in an appropriate format, I attach them to this document again.

We very much hope that the additions to the manuscript and the additional descriptions in this letter will help to better describe our approach so that we can meet the requirements of the reviewers and the editor. We thank again all reviewers and the editor for their help.

Best wishes!

Table1a: regression models for fixation duration

	fixation duration (s)					
	model 1			model 2		
**fixed effects**	estimate	SE	X² (p)	estimate	SE	X² (p)
intercept	2.60	0.48	17.88 (<.001)	2.55	0.51	16.41 (<.001)
BMI	-0.98	0.56	2.92 (0.087)	-0.45	0.49	0.00 (1.000)
energy density	-0.64	0.25	6.29 (0.012)	-0.46	0.23	3.93 (0.047)
companion (partner)	-0.67	0.59	1.29 (0.256)	-0.98	0.60	1.73 (0.189)
gender	-0.85	0.49	2.85 (0.091)	-0.80	0.52	1.46 (0.227)
BMI x energy density	0.96	0.56	2.76 (0.097)	-	-	-
**random effect**	variance	SD		variance	SD	
participant	0.39	0.62	-	0.48	0.69	-
residual	0.71	0.84	-	0.71	0.84	-

Table1b: regression models for visit duration

	visit duration (s)					
	model 1			model 2		
**fixed effects**	estimate	SE	X² (p)	estimate	SE	X² (p)
intercept	3.02	0.59	18.25 (<.001)	2.95	0.62	16.98 (<.001)
BMI	-1.17	0.70	2.68 (0.102)	-0.56	0.56	0.00 (1.000)
energy density	-0.77	0.37	4.22 (0.040)	-0.54	0.33	2.57 (0.109)
companion (partner)	-0.54	0.71	0.59 (0.443)	-0.89	0.71	0.62 (0.430)
gender	-0.79	0.60	1.70 (0.192)	-0.74	0.63	0.33 (0.564)
BMI x energy density	1.09	0.77	1.85 (0.174)	-	-	-
**random effect**	variance	SD		variance	SD	
participant	0.32	0.57	-	0.43	0.65	-
residual	1.61	1.27	-	1.61	1.27	-

Table2a: regression models for fixation count

	fixation count					
	model 1			model 2		
**fixed effects**	estimate	SE	X² (p)	estimate	SE	X² (p)
intercept	7.82	1.79	13.82 (<.001)	7.62	1.83	12.17 (<.001)
BMI	-3.23	2.10	2.27 (0.132)	-.170	1.71	0.00 (1.000)
energy density	-1.86	1.03	3.20 (0.074)	-1.31	0.92	1.97 (0.160)
companion (partner)	-1.14	2.17	0.28 (0.599)	-1.97	2.13	0.00 (1.000)
gender	-1.47	1.81	0.65 (0.419)	-1.33	1.86	0.00 (1.000)
BMI x energy density	2.65	2.20	1.41 (0.235)	-	-	-
**random effect**	variance	SD		variance	SD	
participant	4.25	2.06	-	4.67	2.18	-
residual	11.96	3.46	-	12.02	3.05	-

Table2b: regression models for visit count

	visit count					
	model 1			model 2		
**fixed effects**	estimate	SE	X² (p)	estimate	SE	X² (p)
intercept	3.12	0.44	24.35 (<.001)	3.09	0.46	24.25 (<.001)
BMI	-.053	0.52	0.99 (0.319)	-0.22	0.42	0.41 (0.522)
energy density	-0.52	0.28	3.40 (0.065)	-0.40	0.25	2.55 (0.110)
companion (partner)	-0.41	0.53	0.58 (0.447)	-0.59	0.53	1.37 (0.242)
gender	-0.81	0.45	2.988 (0.084)	-0.77	0.46	2.97 (0.085)
BMI x energy density	0.55	0.57	0.85 (0.357)	-	-	-
**random effect**	variance	SD		variance	SD	
participant	0.19	0.44	-	0.24	0.49	-
residual	0.88	0.94	-	0.87	0.93	-
