# Supplementary material for: Visual attention towards food during unplanned purchases – A pilot study using mobile eye tracking technology
Source: PLoS One. 2021 Mar 4;16(3):e0247755. doi: 10.1371/journal.pone.0247755 (PMC7932502; doi:10.1371/journal.pone.0247755)
Supplement: Attachment — Submitted filename: comments & rebuttals.docx [file pone.0247755.s002.docx]

| **Reviewer 1** | **Comments and rebuttals** |
| --- | --- |
| 1. My biggest concern relates to sample size. There are two weight classifications (high and low BMI), with less than 10 subjects in each category. This is a low number unlikely to be supported by an ex ante power calculation. I understand that obtaining a large number of participants in a real setting, particularly subjects with high BMI, can be somewhat of a challenge. But any statistical analysis based on such few subjects would, at best, be highly preliminary. I would encourage the authors to increase their sample size significantly. | This is indeed one of the most discussable issues and we are aware of it. At this point we also want to disclose that we cannot refute this argument, since it is not possible to re-recruit or simply increase the sample.  We already used several indications and explanations to point out the limitations regarding the sample. This fundamental limitation was also the reason for naming the study a pilot study. Furthermore, we added Limitations in the limitation section that pointed out the limited scope and validity of findings regarding the small sample.  We gratefully accept the advice of the reviewer and now additionally point out that results based on such a small sample size are of course highly preliminary.  Increasing the sample size would be the most appropriate solution to this problem, but unfortunately, we cannot do that under the current circumstances (also considering the influence of the corona virus). At this point we do point out that further studies have to confirm the results from our study using larger samples. |
| Subjects are classified into two weight categories based on self-reported measures of height and weight. Since BMI classification is a main component of this study, relying on self-reported measures is not ideal. If the authors are to increase sample size, I suggest they look into ways to collect weight and height measures during the experiment; for example, experimenters could collect these measures at the end of the last shopping trip, right before payment. | In general, we agree with the reviewer, but there are some particularities to consider. The contact to the participant took place only in the supermarket. Unfortunately, there was no protected area that would have allowed enough privacy for an appropriate and respectful measurement of height and especially weight of the participants. Measuring the physical characteristics of the participants in public would thus have been a fundamental argument, which, from the participants' point of view, would have spoken against participation in the study.  In retrospect, the fundamental recruitment problems and the restrained willingness to participate in the study lead us to accept this aspect as the right way forward. Only in this way were we able to always give the participant a pleasant feeling while participating in the study.  Nevertheless, we have added a paragraph in the methods section, which refers to the specifics of the study. |
| I find the description of experimental procedures to be exceptionally sparse. When was the eye tracking data collected? Was the same weeks used for all subjects? (e.g. eye tracking data was collected for all subjects in weeks 2 and 4), Did subjects knew they would be using eye tracking glasses during those trips beforehand? | The data was collected in the time between January to May 2017. The same weeks were used for each participant (week 1 and 4).  Subjects knew they would wear eye tracking glasses.  We added additional information regarding the questions of the reviewer in the materials and methods section. |
| How many online questionnaires were collected by subject and when were these implemented?  What information did they collect besides socio-economic characteristics? This is important because the type of questions could prime subjects and affect their subsequent purchasing behavior. For example, it would make a big difference if the self-reported weight and height measures were collected at the beginning or at the end of the experiment as asking for subjects’ weight might prime them towards specific food products or product quantities. | All participants were invited to fill in two online questionnaires. The questions were asked after shopping trip 1 and before shopping trip 2.  There were also some psychological scales asked. These are not part of the study.  The duration as well as the sequence of the different surveys of the study and the intervals between them were standardized for all participants.  Questionnaires were positioned between both shopping trips in week one and in week 3 with enough time between them, in order to avoid interference.  In order to make the study procedure more transparent we have added additional information (see figure 1). |
| Who are the participants? The authors mentioned that socio-economic characteristics were collected in the online survey (e.g. gender, age, income); however they do not provide any description of the sample population. I find it a significant omission for an experimental paper to not present a summary of the demographic profile of the sample. This is not necessarily a criticism of the experiment procedures, but rather the written summary of the procedures. I suggest the authors provide a table with a summary of the demographic profile for all the sample and by BMI category (normal weight and overweight/obese). | Thank you for this suggestion. We have included additional socio-demographics for the sample and each weight category in table 1. |
| Each participant received a 50 € voucher as compensation fee at the end of the experiment. Was there another incentive throughout the experiment? If subjects knew they would be receiving the voucher for completing the study, there was no incentive for them to truthfully report their preferences during each shopping visit (i.e. while making shopping lists and purchases). The compensation should have been split into weekly payments. | There was no other voucher throughout the study.  We also discussed different ways of incentivization in our study team. Since we did not want to lose anyone from the small sample during the study, it seemed to us that the best way to do this was to offer a full incentive at the end of the study. In addition, we did not want to have additional weekly money interfere with their usual shopping trip behavior (e.g. buy additional food they usually wouldn’t be because it’s too expensive). |
| Why was participation restricted to households with 2-5 members?  Were participants shopping by themselves? This is important as the presence of a second individual might steer participants toward specific products. For example, Papoutsi et al. (2013) show that children’s pestering power strongly affects parents in making unhealthier food choices. I suggest the authors use data from the online questionnaires (e.g. Who joined the trip?) to control for the number of people in the shopping trips as well as household size. | As shopping behavior differs significantly between persons from single households and families, we have excluded single households from the survey. Similarly, large families with more than five persons are characterized by a particularly different shopping behavior. In principle, this was intended to ensure greater homogeneity between the basic conditions of purchasing. There were no differences between the weight status groups regarding the average household size.  We are also very thankful for the suggestions regarding the shopping companions. We added further information and calculated and tested for differences regarding weight and energy density. Especially the frequency of accompanying partners differ between the weight status groups. We of course discuss this finding in the discussion section. |
| Subjects were asked to write a weekly shopping list and collect grocery receipts for four weeks. When were the receipts and lists collected by the experimenter? If these were collected at the end of the experiment, subjects had the opportunity to revise their shopping lists as much as they wanted. Also, there is the possibility that after 1-2 weeks subjects got an idea of the purpose of the experiment and started adjusting their behavior. I suggest the authors control for week effects. | The self-collected shopping lists and receipts were handed in to the study staff after four weeks. A study that relies on the recording and collection of data by the subject must be confident that the subject can do his or her job. To ensure this, the study team prepared and trained the participants intensively for their tasks. The participants have received oral and written instructions for independent tasks.  The shopping lists were hand written and the authors are unsure why the reviewer would assume/hypothesize that the participants write false shopping lists. However, the study team also subsequently checked the data for irregularities and anomalies. In order to follow the suggestions of the reviewer we also controlled for the time and compared both weeks for the shopping trips. Results can be found in table 2. The results show no differences between purchases regarding weight status groups and energy density groups. |
| The results in Table 1 and Table 2 should be split by BMI and energy density. For example, I would like to see the number of LC and HC unplanned purchases for each BMI category, same for all statistics reported in Table 2. This might cause the significant effects in Table 2 to disappear due to the small sample size, which highlights the need to collect more data. | The reviewer is absolutely right. The analysis is designed to control the potential influences by the group comparisons for the independent variables. To be able to calculate statistics that are at least sufficiently reliable, the independent variables were analyzed isolated. Since the use of statistical analyses should also be appropriate to the sample, an (ex post) analysis of the independent variables in combination seems not to be appropriate.  The number of LC and HC unplanned purchases for each BMI category can be found in Table 1. |
| Throughout the paper, there is no mentioning of the type of statistical tests used (t-tests, one or two-sided, etc.) when statistical analysis is performed. This information needs to be clarified, particularly in Tables and Figures. The authors refer to p-values in Table 2 and Figure 2– what are the statistical tests? The authors should include the relevant missing information in the table notes and figure captions so that they are self-contained. | We thank the reviewer for this valuable advice. All relevant information has been added in the text, in table notes and in figure captions. |
| The analysis is generally lacking in statistical rigor. The results are primarily based on pairwise-comparison tests and I think there are several important factors that need to be controlled for using model specifications/regressions. For example, I would strongly encourage the authors to explore whether the results vary over time (by week); are the findings consistent if one looks at early purchases (e.g. first 2 weeks) vs. later purchases (2 latest). The authors should also control for time-of-the-day effects and individual characteristics such as hunger level, gender, household size, income, eating habits. | We fully agree with the reviewer under the assumption that one has a sufficiently large sample and also collected the corresponding data. Both are only partly true for this study and therefore some compromises have to be made. The selection of relevant control variables was based on current theoretical and empirical findings. The reviewer is nevertheless right: There are other possible influencing variables such as time-of-the-day effects or individual characteristics. However, in the literature, these variables are attributed a negligible influence since they are not directly connected with visual attention. And this is precisely why we have not considered them. For some of these variables (time-of-day effects or further eating habits) no data were collected. |
| Related to my previous comment, the authors are using the observations (unplanned purchases) made by the same individual in different weeks as independent observations. I suggest they consider using models for panel data that cluster the standard errors at the individual level and control for all factors described in the previous comment. They can also test for interaction effects between weight status and energy density using these models rather than ANOVA tests (or complement both). | Since the question of the appropriate form of analysis was raised several times, we followed the reviewer's suggestions and calculated linear mixed effect models for the eye tracking variables. Thereby, subjects were included as random intercept effects in order to include potential effects based on repeated measurements. All other variables were included as fixed effects. We added results, new interpretations and a description in the methods section. We have also addressed concerns about case numbers and specified models that include important control variables. And finally, we took care not to over-fit the model in terms of number of cases. |
| The authors compare attention bias towards the bought product and the labels. What information was provided in the shelf labels? If the only information provided was the product price, a possible explanation for the lower amount of time spent looking at the labels compared to the product could be the familiarity with this attribute. It is reasonable to think that since individuals compare product prices on a regular basis, they might not need as much time to process price information. I think the authors could make more use of the eye tracking data by creating AOIs for the product labels instead of shelf labels. They could explore whether subjects fixate longer on calorie content or health claims such as low-fat, sugar reduced, fat-free, etc. and relate this to unplanned food choices. | Shelf labels provided information about price, discounts, sustainability and brands. The variety of information on the signs was quite high. Also, the complexity of the information on the signs was quite heterogenic. Therefore, it cannot be said with certainty that the lower information density of the shelf signs automatically led to less attention.  Furthermore, the basic idea was not a comparison between products and labels, that was just a control. We wanted to see whether there was possibly also an increased perception of the neighboring products or not.  Further information such as the exact content of the shelf labels was not gathered. Investigations of their influence might also be interesting but were not part of our study.  Additional information regarding this topic have been added in the discussion section. |
| It would have been interesting to see more results related to heterogeneity of unplanned purchases/visual attention of individuals who were more/less hungry according to the scale the authors collected. There is a vast literature on how hunger might produce different effects, and this paper could have something to say about this. | Yes, that’s true. Unfortunately, we still have to cope with this small sample and cannot implement all possible influences into our models. Nevertheless, hunger has been controlled for in table 2 and showed no significant difference between the groups of comparisons. |
| The relationship between visual attention and food choice across BMI categories is an important contribution that has been highly understudied. Only few studies that have examined such distributional effects (Segovia et al. 2019). This is worth stressing more in the paper. | We thank the reviewer for this helpful suggestion. We added some more information including the paper in the theoretical section of the manuscript. |
| The authors should be consistent in the terminology used for BMI categories, sometimes they refer to the groups as high vs. low BMI, sometimes as BMI<25 vs. BMI>25, and other times as normal weight vs. overweight/obese. | We adapted the uniform naming of the groups throughout the manuscript. |
| Are there underweight subjects in the sample? (BMI< 18.5). If so, they should not be part of the normal weight group. | There are no underweight participants in the sample. We added this information to the description of the study sample (in the result section). |
| Page 15, lines 357-358: sentence makes no sense. Please edit. | The sentence has been edited. |
| The limitations of the study can be discussed in the conclusions section, no need for a separate section. | We added the limitations to the conclusions section. |
|  |  |

| **Reviewer 2** | | **Comments and rebuttals** |
| --- | --- | --- |
| The experiment design and hence the findings suffer from a major issue due to the product arrangement in a supermarket. As indicated in Lines 313-315, if the LC foods are offered in the first section, while the HC foods are located in the later sections. This gives a basis to consider that consumers may face less time pressure or constraints in choosing LC foods (i.e., beginning of the shopping experience) and more time pressure/constraint in choosing HC food (i.e., toward the end of the shopping experience). Because of this arrangement, consumers may spend more time and pay more visual attention to LC foods, while paying less visual attention to HC foods. In other words, we don’t know for sure whether the differences in visual attention between HC and LC foods (and across BMI groups) were caused by the product arrangement or the differences in energy density that the authors tried to identify in the study. Generalizing statement such as “There was a tendency across all participants to look at low energy density food longer and more often.” (Lines 12-13) need to be interpreted with caution. The authors may want to acknowledge this as a major limitation of this study. | | Yes that’s absolutely true. We thank reviewer two for this very good comment. We will add some more thoughts about this issue in the conclusions section. |
| Lines 283-287: The authors compared fixation duration between low BMI participants for LC, high BMI participants for LC, and low BMI participants for HC. From my point of view, the authors should conduct a complete between-group and within-group comparison to capture the interaction between BMI and energy density on all VA measures. Using fixation duration as an example, between-group comparison should be conducted for a) between low BMI participants’ fixation duration for LC foods, and high BMI participants’ fixation duration for LC foods, b) between low BMI participants’ fixation duration for HC and high BMI participants’ fixation duration for HC. Within group comparison should be conducted for a) between low BMI individuals’ fixation duration for LC and HC, b) between high BMI individuals’ fixation duration for LC and HC foods. | | We thank the reviewer for this note. In the previous version of the manuscript only the significant ANOVAS of the between groups were reported. We now hope that the regressions have enabled us to fulfill the reviewer's wish. |
| The authors may consider adding a table reporting the demographic variables comparison between the low and high BMI groups as we know BMI is closely related to individual characteristics such as age and income. This information is completely missing in the current version. Demographic information is particularly important for this study because the sample size is very small. | | We thank reviewer 2 for this suggestion. We added additional information regarding social demographics in table 1. |
| Section 2.4. The authors identified consumers’ unplanned purchases by checking their grocery receipts for the past four weeks. As shoppers ourselves, we know perfectly that there are times when last-minute items pop up when we are already at the store, even with a shopping list. Since this is a major variable of interest and used to test two of the three hypotheses, I have concerns about the reliability of this procedure. Please comment. | | That's a good point. We have been thinking about this point for a long time and it was clear to us quite early on that there will not be a 100% solution.  Basically, this depends of course on the definition of unplanned purchase behavior. In our study, we have chosen a simple and thus quite reliable definition and measurement. All purchases that were not previously planned by writing them down on the shopping list were defined as unplanned purchases. So, in fact, it made no difference to our definition when the idea or decision to buy was made during the shopping trip. And thus, the procedure was quite reliable. We added this description in the manuscript. |
| Section 3.1. The number of participants in this study is a concern. I am well aware of the difficulties associated with implementing eye tracking studies, especially at a grocery store. However, without reporting the full picture of the visual attention variable statistics and demographic characteristics, it is difficult to make a judgement about the reliability of the results. | | We understand the concerns of the reviewer and hope that the additional information (see table 1 and 2) is sufficient to get a better picture of the data. |
| 129-131: You are listing three different measures - maintained attention, counts for fixations, and counts for visits. Then, in the next sentence, you are saying both (referring to two) measures are acceptable measures to be included in the analysis. I have no doubt that visual attention variables are relevant in such analyses. However, there are important distinctions between fixation counts (within an AOI or across AOIs) and AOI visits. One can measure fixation counts within an AOI during a single or multiple visits. The other measure, visit counts, can be used (e.g., comparing two different AOIs) without incorporating any fixation counts. Please clarify the exact measure that you are using. Section 2.3. By “maintained attention towards food” are you referring to fixation duration as used in Tobii Pro manuals? | | At this point there is a misunderstanding. We meant, of course, that there are four different measurements. We distinguish between the duration (a time someone looked at sth.) in seconds and the number of counts (how often someone looked at sth.). Both measurements can be recorded for fixations and visits.  We measured fixation duration as the time an AOI was fixated (cumulated for multiple fixations). We measured visit duration as the time the view was over an AOI. This includes fixations and saccades within an AOI (cumulated for multiple visits). We measured the fixation counts as the cumulated number of fixations within one AOI (cumulated for multiple fixations). And we measured the visit counts as the cumulated number of visits within one AOI (cumulated for multiple visits).  We changed the description of the measurements in the manuscript. |
| Section 3.3. Heat map grid:  o Plot C – Duration for fixations and visits. Is this the duration of the total number of fixations and total number of visits within an AOI/grid? Again, the number of fixations and number of visits are conventionally defined differently. Unless you are assuming different definitions in this particular study. Please explain.  o Plot D – Again, I am not sure how the number of fixations and visit can be measured as one variable. One can visually attend to (visit) an AOI, and fixate multiple times during that one visit. Please clarity.  o How was the irregular size of the product spaces on real store shelves mapped into the current grid system? If the space above or below the purchased product includes more than one product, how were situations like those handled. I would be interested in examining the actual heatmaps and the manual mapping of the fixations and the image of the purchased product shelves. | | We have hopefully added a better and more precise description in the manuscript which helps better understand our measurements.  The different relations are not shown in the heat maps. That’s why we call it standardized heat maps. Size, color and shape of the products are no longer relevant; all products have been reduced to a uniform shape. To be able to implement this procedure uniformly, corresponding coding rules for the creation of heat maps were defined. The standardization of the heat maps was also part of the coder training and the good reliability test.  Attached you will find an example of the standardized heatmap (A) and the original picture (B).   |
| Section 2.1. When was the experiment conducted? Was the recruitment done right at the store? | | The data was gathered in early 2017. The exact time was added in the manuscript.  The recruitment was done one week before the first eye tracking measurement. The new figure 1 shows the study procedure by time. |
| Section 3.2. o What is the measure of shelf height in Table 2? o Is the unplanned purchase duration (in seconds) measured when taking the item from the shelf and placing in the cart? o It would be useful to include a column that defines these variables. o First time use of VAS in table 2. Please explain what it means. | | Since not all shelves in the supermarket are the same size and we could not measure all the shelves, we worked with the shelves' inserts. For us, the relative height of the shelves resulted from the relation between the shelf on which the product stands and the maximum number of shelves in the cabinet. If the product is placed on the second shelf and there are 8 in total on the shelf, this gives a value of 0.25 as the relative cabinet height. This measure is therefore more of a proxy for whether something is more likely to be at the top or bottom of the shelf. We added a description under the corresponding table.  Yes, unplanned purchase duration is the time it took from the first fixation to the selection of the product.  VAS means visual analogue scale. We added a description in the notes under the corresponding table. |
|  |  | |

| **Reviewer 3** | **Comments and rebuttals** |
| --- | --- |
| Line 9: attention bias. If attention is influenced by energy density and weight status, why is it necessarily a bias? | It is true that a difference in visual attention is not necessarily a distortion. That is why we have changed the corresponding section in the text. Instead, we now speak of differences in visual attention. |
| Line 13: Can you explain more about the interaction: “Interaction effects between weight status and energy density for fixation and visit duration were found” | We have deleted this part because it no longer matches the findings of the updated analyses. |
| Line 22, can you better define unplanned purchases? | In our study, we have chosen a simple and thus quite reliable definition and measurement. All purchases that were not previously planned by writing them down on the shopping list were defined as unplanned purchases. We added this description in the manuscript. |
| Line 33: what about food purchases, how many are unplanned? | We did not add the corresponding numbers to the manuscript because the focus was not on them. The exact number or share can only be estimated. But it is clearly below 50%. Probably even below 20%. |
| Line 52: initial orientation? What do you mean? | Initial orientation is a term that comes from experimental settings and denotes the first subconscious affections towards stimuli. It answers the question of where to look first. |
| Line 55: “Gearhardt et al. (2012) showed a decreased initial attention towards fried food for participants with higher BMI”, decreased compared to what? | We added additional information to the introduction section. |
| Line 69: you hypothesize that is differs, but can you be more specific? Do you think VA will be higher or lower? | We have limited ourselves to the postulation of differences, since the theory and the empirical findings of previous studies did not allow a clear statement in favor of one side. |
| Line 87: In what way is there in interaction? What is the direction? Can you be more specific of your H3? | New analysis do not show any interactions any longer. |
| How did you know which purchases were unplanned? | In our study, we have chosen a simple and thus quite reliable definition and measurement. All purchases that were not previously planned by writing them down on the shopping list were defined as unplanned purchases. |
| Line 36: why no constraints? What do you mean by this? | Many studies that have taken place under real-world conditions (e.g. Wang et al. 2018) require one thing: You have to buy, choose, and often consume something. This pressure alone, which does not exist in any real situation, diminishes the ecological validity. In our study, no one was at any time induced or even tempted to make a purchase. The purchases that took place took place without pressure and under real conditions. |
| Line 127: what is the measurement error etc. | The average measurement error of the Tobii glasses 2 was between 0.6° - 1.2° of visual angle. We added this information to the eye tracking section (2.2.). |
| Line 132: “first fixation”, do you mean “time to first fixation”? | Yes, we mean of course time to first fixation. We corrected this in the text. |
| Line 149-150: what is the disadvantage using no overlap? What about peripheral vision? What about eye tracking errors? Should it not be accounted for a bitmore by making the AOIs a bit larger than the products themself? | Yes, that's a fair question. In any experimental design we would always work with overlaps, because there is often enough space between the stimuli. But in our supermarket, there was often no space between the products. By overlapping the AOIs, we would then have counted the values for two AOIs when looking at their edges. Subsequently, we would never have been able to decide on which AOI the fixation was now on. Moreover, due to the different distances to the products, we would have had to work with various AOI overlaps in order to adequately consider the error rates. This would have made working for the coders much more difficult, if not impossible. |
| Was there a relation between attention and choice and if so, was this influenced by the weight status? | We cannot say anything about the relationship between attention and choice. To do so, we would have had to consider decision-making processes that did not lead to a purchase. Unfortunately, we do not have any data on this.  Table one shows no differences for unplanned purchase behavior regarding weight status. New analysis do not show any relations between weight status and maintained visual attention. |
| Line 177: “ During these trips, 16 participants showed unplanned purchase behavior and three participants showed no unplanned purchase behavior at all” . How do you know it is unplanned? How did you measure this? How did you define it? | In our study, we have chosen a simple and thus quite reliable definition and measurement. All purchases that were not previously planned by writing them down on the shopping list were defined as unplanned purchases. So we only had to compare the shopping lists with the receipts to identify the unplanned purchases. |
| Line 218 identi-fied: typo  The product in the middle is row P. Did each isle have 7 shelves? What if a chosen product is on the bottom shelf, then there is no P-1, P-2, P-3. It is not clear how you addressed this. | Yes, the chosen product in the middle is P. You are absolutely right. If a chosen product is at the bottom shelf, then there is no P-1, P-2, P-3. The same for the top shelf. This also applies to the product on the far left or right of the shelf. |
| Line 222: “The product one row above”. Do you mean one shelf higher? | Yes. We thank the reviewer for this comment and edited the sentence in the manuscript. |
| Line 261: “ normal weight noticed the food of their choice earlier and looked longer at it than participants with overweight or obesity”. Due to this result, you confirm H1. However in H1 you only state that the VA would differ. Did you have a specific hypothesis how it would differ? In which case would it be higher? Does the results match this hypothesis? | By writing “ normal weight noticed the food of their choice earlier and looked longer at it than participants with overweight or obesity” we also imply that there is a difference between both groups. But we changed this phrase in the text to make it more appropriate. |
| Line 235-236: “ While information labels and price tags were on average focused by three spontaneous purchase decisions (M = 3.1, SD = 5.1), the products themselves were focused more than twice as often (M = 7.3, SD = 11.9).” This sentence is not clear. The labels and price tags were focused by purchase decisions? Please rephrase. | We rephrased this sentence. |
| Check, sometimes VA and sometimes in full “Visual attention”. Please be consistent in the use of the abbreviations. | Thank you, we now use VA consistently throughout the whole document. |
| Line 305: “Participants with normal weight made 76% of the unplanned purchases”. Again, I wonder how you defined unplanned purchases and how this was measured. | This point has already been discussed above. |
| What are the results for people with overweight? Does your study provide insight in how we can help them to attend to energy information? | Since the new analysis show no influence of BMI we really do not know whether people with overweight are particularly at risk. But if this is the case, it is always helpful to have a healthy and relevant set of products that can be used in case of an unplanned purchase. |
| What about planned food purchases? | Planned purchases are also an interesting research area, but they were not within the scope of our study. And consequently, there is no data available on planned purchases using mobile eye tracking technology. Furthermore, planned purchases describe a completely different situation. There is often a relevant set of products from which a pre-selection is already made. So the decision making process takes place when writing the shopping list. The visual attention at the POS thus becomes insignificant. |
