# Supplementary material for: Visual attention towards food during unplanned purchases – A pilot study using mobile eye tracking technology
Source: PLoS One. 2021 Mar 4;16(3):e0247755. doi: 10.1371/journal.pone.0247755 (PMC7932502; doi:10.1371/journal.pone.0247755)
Supplement: Attachment — Submitted filename: PLOS One reviewR2.docx [file pone.0247755.s003.docx]

|  |  | *Most of the comments have been addressed and the manuscript has been largely improved.* |
| --- | --- | --- |
| The experiment design and hence the findings suffer from a major issue due to the product arrangement in a supermarket. As indicated in Lines 313-315, if the LC foods are offered in the first section, while the HC foods are located in the later sections. This gives a basis to consider that consumers may face less time pressure or constraints in choosing LC foods (i.e., beginning of the shopping experience) and more time pressure/constraint in choosing HC food  (i.e., toward the end of the shopping experience). Because of this arrangement, consumers may spend more time and pay more visual attention to LC foods, while paying less visual attention to HC foods. In other words, we don’t know for sure whether the differences in visual attention between HC and LC foods (and across BMI groups) were caused by the product arrangement or the differences in energy density that the authors tried to identify in the study. Generalizing statement such as “There was a tendency across all participants to look at low energy density food longer and more often.” (Lines 12-13) need to be interpreted with caution. The authors may want to acknowledge this as a major limitation of this study. | Yes that’s absolutely true. We thank reviewer two for this very good  comment. We will add some more thoughts about this issue in the  conclusions section. | *Basically, this major comment was not addressed and was not addressable at this stage because this issue should have been considered in the experimental design stage. But thank you for recognizing this as one of the limitations of this study in the Conclusions section.* |
| Lines 283-287: The authors compared fixation duration between low BMI participants for LC, high BMI participants for LC, and low BMI participants for HC. From my point of view, the authors should conduct a complete between group and within-group comparison to capture the interaction between BMI and energy density on all VA measures. Using fixation duration as an example, between-group comparison should be conducted for a) between low BMI participants’ fixation duration for LC foods, and high BMI participants’ fixation duration for LC foods, b) between low BMI participants’ fixation duration for HC and high BMI participants’ fixation duration for HC. Within group comparison should be conducted for a) between low BMI individuals’ fixation duration for LC and HC, b) between high BMI individuals’ fixation duration for LC and HC foods. | We thank the reviewer for this note. In the previous version of the manuscript  only the significant ANOVAS of the between groups were reported. We now hope that the regressions have enabled us to fulfill the reviewer's wish. | *Thank you for addressing this comment by conducting additional regression analysis. However, I do have some questions regarding the interpretation of the regression results.*  *First, what does the intercept measure? Taking Fixation Counts as an example. How would you interpret the estimate of 10.19 (P<0.05)? Applying the standard logic of interpretating the intercept in linear regression, the intercept of 10.19 essentially means that the average fixation counts of the sample is around 10 times (i.e., the fixation counts of a male shopper with zero BMI, without companion, purchasing a product with zero ED is about 10). The fixation counts in Figure 3 for low ED is <6 and about 4 for high ED (lower panel). So the regression results are consistent with Figure 3 in terms of statistical significance.*  *This estimate is much higher than the fixation counts plot in Figure 3 even taking into consideration of the negative impact of companion. With a companion reduces almost three fixation counts. The negative impact of ED is statically significant but marginal as the coefficient is very small.*  *My guess is that the variable BMI (exact) and energy density (exact) are not binary variables. They are the exact number of (self-reported) BMI of each participant and the exact ED of each product purchased. Please correct me if I am wrong.*  *Therefore, my suggestion would be why not run the regression model using BMI and ED dummies (say, high BMI=1, high ED=1). Then the intercept actually captures the average fixation counts (fixation duration, visit counts, visit duration) for a male shopper with low BMI, without companion purchased low ED product. The interpretation of the intercepts as well as other coefficients in all four maintained attention models would be neat, interesting and comparable with your plots in Figure 3.*  *In addition, you mentioned in the revised manuscript that adding interaction terms do not improve model (Lines 376-382). With my suggestion of using dummies, I would like you to try the interaction terms of BMI and ED dummies again to see if the interaction term is significant or not. Combining the coefficients of BMI, ED and the interaction term of BMI and ED, will provide you the effect between and within group compaction that I mentioned.* |
