# Supplementary material for: Visual attention towards food during unplanned purchases – A pilot study using mobile eye tracking technology
Source: PLoS One. 2021 Mar 4;16(3):e0247755. doi: 10.1371/journal.pone.0247755 (PMC7932502; doi:10.1371/journal.pone.0247755)
Supplement: Attachment — Submitted filename: Rebuttals and Revisions.docx [file pone.0247755.s004.docx]

**Comments to the reviewers**

We thank the reviewers for their helpful and inspiring advices. We believe with your help we were able to improve the manuscript once again.

Before we address the questions of the reviewers in the table below, we would like to point out an error in the old manuscript. In the old version of the manuscript, the name of the cut-off point for the division into high and low calorie foods was incorrectly defined. The cut off was labeled as 100kcal/100g. The correct label for the value is 260kcal/100g. The 260kcal/100g mark is the average value of the energy density of the bought products in the current study and can roughly be seen as the transition point from high calorie food to lower energy density foods (Bechtold, 2014, S.5). We added this information in the paragraph in the manuscript. The correction of the label has no effect on the calculations. The calculations have already been performed with the correct values and only the label was incorrectly defined. We must apologize for this mistake and hope that the changes will meet the demands of the reviewers.

Below you will find our comments and answers to your questions.

| **Comments reviewer 2** | |
| --- | --- |
| Most of the comments have been addressed and the manuscript has been largely improved.  Basically, this major comment was not addressed and was not addressable at this stage because this issue should have been considered in the experimental design stage. But thank you for recognizing this as one of the limitations of this study in the Conclusions section. | We want to thank reviewer 2 for this positive feedback.  Unfortunately, there was no way to manipulate the arrangement in the supermarket and collect data in the real world. Collecting data in the real world setting also means having to make compromises. That’s why we controlled for this influence and discussed this issue at the end of the manuscript. |
| Thank you for addressing this comment by conducting additional regression analysis. However, I do have some questions regarding the interpretation of the regression results.  First, what does the intercept measure? Taking Fixation Counts as an example. How would you interpret the estimate of 10.19 (P<0.05)? Applying the standard logic of interpretating the intercept in linear regression, the intercept of 10.19 essentially means that the average fixation counts of the sample is around 10 times (i.e., the fixation counts of a male shopper with zero BMI, without companion, purchasing a product with zero ED is about 10). The fixation counts in Figure 3 for low ED is <6 and about 4 for high ED (lower panel). So the regression results are consistent with Figure 3 in terms of statistical significance.  This estimate is much higher than the fixation counts plot in Figure 3 even taking into consideration of the negative impact of companion. With a companion reduces almost three fixation counts. The negative impact of ED is statically significant but marginal as the coefficient is very small.  My guess is that the variable BMI (exact) and energy density (exact) are not binary variables. They are the exact number of (self-reported) BMI of each participant and the exact ED of each product purchased. Please correct me if I am wrong.  Therefore, my suggestion would be why not run the regression model using BMI and ED dummies (say, high BMI=1, high ED=1). Then the intercept actually captures the average fixation counts (fixation duration, visit counts, visit duration) for a male shopper with low BMI, without companion purchased low ED product. The interpretation of the intercepts as well as other coefficients in all four maintained attention models would be neat, interesting and comparable with your plots in Figure 3.  In addition, you mentioned in the revised manuscript that adding interaction terms do not improve model (Lines 376-382). With my suggestion of using dummies, I would like you to try the interaction terms of BMI and ED dummies again to see if the interaction term is significant or not. Combining the coefficients of BMI, ED and the interaction term of BMI and ED, will provide you the effect between and within group compaction that I mentioned. | Reviewer 2 is right. The variables BMI (exact) and energy density (exact) are not binary variables. They are the exact number of (self-reported) BMI of each participant and the exact ED of each product purchased. We decided to use the exact numbers instead of dichotomized grouping variables which may already contain an inaccuracy due to the ‘artificial’ grouping.  Nevertheless, we would like to fulfill the reviewer's wish and calculate the regressions again with the dichotomized variables for BMI and energy density.  The results for this analysis can be seen in tables 1a, 1b, 2a, and 2b. All tables are attached (outside the textbox) below.  We have calculated two models for each eye tracking measurement (FD, VD, FC, VC). The models have been calculated including variables for gender, the presence of a companion, BMI (dichotomized) and energy density (dichotomized) as fixed effects. We additionally included the interaction between BMI and energy density as a fixed effect in model 1. The individual level was included as random effect in all models.  The additional analyses are leading to two results. 1.) Even with dichotomized variables we cannot see any significant interaction effect for any eye tracking measurement.  2.) Since the new calculations with dichotomous variables did not result in a significant improvement of the regression models, we decided to keep the first models with continuous variables.  One last information about the interpretation of the intercepts of the fixed effect in random effect models. The estimator of the fixed effect for the random effects models is the appropriate estimate for the intercept of the random-effects. Below (inside the textbox) you will find the output of the estimates for the ‘individual intercepts’ for the random effects for the regression model calculated for FD. In one last step, one can calculate the mean of these values and will get the value for the intercept.   |

**Table1a:** regression models for fixation duration

|  | **fixation duration (s)** | | | | | | |
| --- | --- | --- | --- | --- | --- | --- | --- |
|  | **model 1** | | | | **model 2** | | |
| **fixed effects** | estimate | SE | Χ² (p) | estimate | | SE | Χ² (p) |
| intercept | 2.60 | 0.48 | 17.88 (<.001) | 2.55 | | 0.51 | 16.41 (<.001) |
| BMI | -0.98 | 0.56 | 2.92 (0.087) | -0.45 | | 0.49 | 0.00 (1.000) |
| energy  density | -0.64 | 0.25 | 6.29 (0.012) | -0.46 | | 0.23 | 3.93 (0.047) |
| companion (partner) | -0.67 | 0.59 | 1.29 (0.256) | -0.98 | | 0.60 | 1.73  (0.189) |
| gender | -0.85 | 0.49 | 2.85 (0.091) | -0.80 | | 0.52 | 1.46 (0.227) |
| BMI x  energy density | 0.96 | 0.56 | 2.76 (0.097) | - | | - | - |
| **random effect** | variance | SD |  | variance | | SD |  |
| participant | 0.39 | 0.62 | - | 0.48 | | 0.69 | - |
| residual | 0.71 | 0.84 | - | 0.71 | | 0.84 | - |

**Table1b:** regression models for visit duration

|  | **visit duration (s)** | | | | | | |
| --- | --- | --- | --- | --- | --- | --- | --- |
|  | **model 1** | | | | **model 2** | | |
| **fixed effects** | estimate | SE | Χ² (p) | estimate | | SE | Χ² (p) |
| intercept | 3.02 | 0.59 | 18.25  (<.001) | 2.95 | | 0.62 | 16.98 (<.001) |
| BMI | -1.17 | 0.70 | 2.68 (0.102) | -0.56 | | 0.56 | 0.00  (1.000) |
| energy  density | -0.77 | 0.37 | 4.22 (0.040) | -0.54 | | 0.33 | 2.57 (0.109) |
| companion (partner) | -0.54 | 0.71 | 0.59  (0.443) | -0.89 | | 0.71 | 0.62 (0.430) |
| gender | -0.79 | 0.60 | 1.70  (0.192) | -0.74 | | 0.63 | 0.33 (0.564) |
| BMI x  energy density | 1.09 | 0.77 | 1.85  (0.174) | - | | - | - |
| **random effect** | variance | SD |  | variance | | SD |  |
| participant | 0.32 | 0.57 | - | 0.43 | | 0.65 | - |
| residual | 1.61 | 1.27 | - | 1.61 | | 1.27 | - |

**Table2a:** regression models for fixation count

|  | **fixation count** | | | | | | |
| --- | --- | --- | --- | --- | --- | --- | --- |
|  | **model 1** | | | | **model 2** | | |
| **fixed effects** | estimate | SE | Χ² (p) | estimate | | SE | Χ² (p) |
| intercept | 7.82 | 1.79 | 13.82 (<.001) | 7.62 | | 1.83 | 12.17  (<.001) |
| BMI | -3.23 | 2.10 | 2.27 (0.132) | -.170 | | 1.71 | 0.00 (1.000) |
| energy  density | -1.86 | 1.03 | 3.20 (0.074) | -1.31 | | 0.92 | 1.97  (0.160) |
| companion (partner) | -1.14 | 2.17 | 0.28  (0.599) | -1.97 | | 2.13 | 0.00  (1.000) |
| gender | -1.47 | 1.81 | 0.65 (0.419) | -1.33 | | 1.86 | 0.00 (1.000) |
| BMI x  energy density | 2.65 | 2.20 | 1.41 (0.235) | - | | - | - |
| **random effect** | variance | SD |  | variance | | SD |  |
| participant | 4.25 | 2.06 | - | 4.67 | | 2.18 | - |
| residual | 11.96 | 3.46 | - | 12.02 | | 3.05 | - |

**Table2b:** regression models for visit count

|  | **visit count** | | | | | | |
| --- | --- | --- | --- | --- | --- | --- | --- |
|  | **model 1** | | | | **model 2** | | |
| **fixed effects** | estimate | SE | Χ² (p) | estimate | | SE | Χ² (p) |
| intercept | 3.12 | 0.44 | 24.35  (<.001) | 3.09 | | 0.46 | 24.25  (<.001) |
| BMI | -.053 | 0.52 | 0.99  (0.319) | -0.22 | | 0.42 | 0.41  (0.522) |
| energy  density | -0.52 | 0.28 | 3.40  (0.065) | -0.40 | | 0.25 | 2.55  (0.110) |
| companion (partner) | -0.41 | 0.53 | 0.58  (0.447) | -0.59 | | 0.53 | 1.37 (0.242) |
| gender | -0.81 | 0.45 | 2.988  (0.084) | -0.77 | | 0.46 | 2.97  (0.085) |
| BMI x  energy density | 0.55 | 0.57 | 0.85 (0.357) | - | | - | - |
| **random effect** | variance | SD |  | variance | | SD |  |
| participant | 0.19 | 0.44 | - | 0.24 | | 0.49 | - |
| residual | 0.88 | 0.94 | - | 0.87 | | 0.93 | - |

| **Comments reviewer 3** | |
| --- | --- |
| The authors have has improved the manuscript. However, a few small questions still remain. | We thank reviewer 3 for this positive feedback. |
| • You mention that you changed attention bias to visual attention. However, this is not adjusted everywhere (for example line 281). Please explain. | We thank the reviewer for the attentive reading and this note. Accordingly, we have revised corresponding parts in the Manuscript. See lines 281-282 and line 462-463. |
| • The product in the middle is row P. Did each isle have 7 shelves? What if a chosen product is on the bottom shelf, then there is no P-1, P-2, P-3. It is not clear how you addressed this. You mention that a product at the bottom shelf, will not have P-1, P-2 etc. How did this impact the generation of Figure 1? | The product in the middle of the standardized heat map is always the bought product (and abbreviated as P).  Not every isle in the supermarket had 7 shelves or was 11 product rows wide. Some isles even had more than 7 shelves and many isles had more than 11 product rows. Thus, the heat maps represent a standardized and reduced section of the shelf walls in the supermarket surrounding the purchased product. The aspect ratio of the heat maps was chosen as landscape format according to human vision.  Average relative shelve height is 0.6 (see table 2). Further measures of dispersion for relative shelf height are: min=0.14, max=1, Q1=0.33, Q3=0.85. Thus, the middle 50% of all bought products have been positioned in the relative shelve height between 0.33 and 0.85. (see Boxplot for relative shelve height).    The results show what we already know from former studies. The majority of the purchased products were found in the middle shelf area, slightly above the middle of the shelves (which is at 0.5). Some measurements were found in the lower and some in the upper areas. Since the values are quite equally distributed over the entire height of the shelves, the effect on the production of the heat maps should be quite small and negligible. |

**References**

Bechtold, A. (2014). Energiedichte der Nahrung und Körpergewicht Wissenschaftliche Stellungnahme der DGE. https://www.ernaehrungs-umschau.de/fileadmin/Ernaehrungs-Umschau/pdfs/pdf_2014/01_14/EU01_2014_M014_M023_-_002d_011d.qxd.pdf
