# Supplementary material for: Visual attention towards food during unplanned purchases – A pilot study using mobile eye tracking technology
Source: PLoS One. 2021 Mar 4;16(3):e0247755. doi: 10.1371/journal.pone.0247755 (PMC7932502; doi:10.1371/journal.pone.0247755)
Supplement: Attachment — Submitted filename: PLOS One reviewR2.docx [file pone.0247755.s005.docx]

| **Previous comments in R1** | **Authors’ response** | **R2 comments** |
| --- | --- | --- |
| Basically, this major comment was not addressed and was not addressable at this stage because this issue should have been considered in the experimental design stage. But thank you for recognizing this as one of the limitations of this study in the Conclusions section. | We want to thank reviewer 2 for this positive feed-back.  Unfortunately, there was no way to manipulate the arrangement in the supermarket  and collect data in the real world. Collecting data in the real world setting also means  having to make compromises. That’s why we controlled for this influence and  discussed this is-sue at the end of the manuscript. | *You did briefly discuss the issue of fixed arrangement at the end of the manuscript. I also read in your response the following “That’s why we controlled for this influence and discussed this issue at the end of the manuscript.” However, my understanding is that you did not control the influence of fixed arrangement in your experiment. Please clarify your statement.*  *One way to control the influence of fixed arrangement would be through randomizing the starting point of your participants’ shopping experiences. For example, if low caloric foods were located in the front sections of the store (and high caloric foods in the back), you could instruct half of your participants to start shopping from the front. The other half of the participants would then start shopping from the back of the store. This would ensure that all of your participants didn’t start by paying attention to and purchasing low caloric foods first, thus controlling for the fixed arrangements. Does this make sense?* |
| Thank you for addressing this comment by conducting additional regression analysis. However, I do have some questions regarding the interpretation of the regression results.  First, what does the intercept measure? Taking Fixation Counts as an example. How would you interpret the estimate of 10.19 (P<0.05)? Applying the standard logic of interpretating the intercept in linear regression, the intercept of 10.19 essentially means that the average fixation counts of the sample is around 10 times (i.e., the fixation counts of a male shopper with zero BMI, without companion, purchasing a product with zero ED is about 10). The fixation counts in Figure 3 for low ED is <6 and about 4 for high ED (lower panel). So the regression results are consistent with Figure 3 in terms of statistical significance.  This estimate is much higher than the fixation counts plot in Figure 3 even taking into consideration of the negative impact of companion. With a companion reduces almost three fixation counts. The negative impact of ED is statically significant but marginal as the coefficient is very small.  My guess is that the variable BMI (exact) and energy density (exact) are not binary variables. They are the exact number of (self-reported) BMI of each participant and the exact ED of each product purchased. Please correct me if I am wrong.  Therefore, my suggestion would be why not run the regression model using BMI and ED dummies (say, high BMI=1, high ED=1). Then the intercept actually captures the average fixation counts (fixation duration, visit counts, visit duration) for a male shopper with low BMI, without companion purchased low ED product. The interpretation of the intercepts as well as other coefficients in all four maintained attention models would be neat, interesting and comparable with your plots in Figure 3.  In addition, you mentioned in the revised manuscript that adding interaction terms do not improve model (Lines 376-382). With my suggestion of using dummies, I would like you to try the interaction terms of BMI and ED dummies again to see if the interaction term is significant or not. Combining the coefficients of BMI, ED and the interaction term of BMI and ED, will provide you the effect between and within group compaction that I mentioned. | Reviewer 2 is right. The variables BMI (exact) and energy density (exact) are not binary variables. They are the exact number of (self-reported)  BMI of each participant and the exact ED of each product pur-chased. We decided to  use the exact numbers in-stead of dichotomized grouping variables which may already  contain an inaccuracy due to the ‘artificial’ grouping.  Nevertheless, we would like to fulfill the reviewer's wish and calculate the regressions  again with the di-chotomized variables for BMI and energy density.  The results for this analysis can be seen in tables 1a, 1b, 2a, and 2b. All tables are  attached (outside the textbox) below.  We have calculated two models for each eye tracking measurement (FD, VD, FC, VC).  The models have been calculated including variables for gender, the presence of a  companion, BMI (dichotomized) and energy density (dichotomized) as fixed effects.  We additionally included the interaction between BMI and energy density as a fixed  effect in model 1. The indi-vidual level was included as random effect in all models.  The additional analyses are leading to two results.  1.) Even with dichotomized variables we cannot see any significant interaction effect  for any eye tracking measurement.  2.) Since the new calculations with dichotomous vari-ables did not result in a significant  improvement of the regression models, we decided to keep the first mod-els with  continuous variables.  One last information about the interpretation of the intercepts of the fixed effect in  random effect models. The estimator of the fixed effect for the random ef-fects models  is the appropriate estimate for the inter-cept of the random-effects. Below (inside the  textbox) you will find the output of the estimates for the ‘indi-vidual intercepts’ for the random effects for the re-gression model calculated for FD. In one last step, one can  calculate the mean of these values and will get the value for the intercept.  Table1a: regression models for fixation duration  fixation duration (s)  model 1model 2  fixed effectsestimateSEΧ² (p)estimateSEΧ² (p)  intercept2.600.4817.88  (<.001)2.550.5116.41  (<.001)  BMI-0.980.562.92  (0.087)-0.450.490.00  (1.000)  energy  density-0.640.256.29  (0.012)-0.460.233.93  (0.047)  companion (partner)-0.670.591.29  (0.256)-0.980.601.73  (0.189)  gender-0.850.492.85  (0.091)-0.800.521.46  (0.227)  BMI x  energy density0.960.562.76  (0.097)---  random effectvarianceSDvarianceSD  participant0.390.62-0.480.69-  residual0.710.84-0.710.84-  Table1b: regression models for visit duration  visit duration (s)  model 1model 2  fixed effectsestimateSEΧ² (p)estimateSEΧ² (p)  intercept3.020.5918.25  (<.001)2.950.6216.98  (<.001)  BMI-1.170.702.68  (0.102)-0.560.560.00  (1.000)  energy  density-0.770.374.22  (0.040)-0.540.332.57  (0.109)  companion (partner)-0.540.710.59  (0.443)-0.890.710.62  (0.430)  gender-0.790.601.70  (0.192)-0.740.630.33  (0.564)  BMI x  energy density1.090.771.85  (0.174)---  random effectvarianceSDvarianceSD  participant0.320.57-0.430.65-  residual1.611.27-1.611.27-  Table2a: regression models for fixation count  fixation count  model 1model 2  fixed effectsestimateSEΧ² (p)estimateSEΧ² (p)  intercept7.821.7913.82  (<.001)7.621.8312.17  (<.001)  BMI-3.232.102.27  (0.132)-.1701.710.00  (1.000)  energy  density-1.861.033.20  (0.074)-1.310.921.97  (0.160)  companion (partner)-1.142.170.28  (0.599)-1.972.130.00  (1.000)  gender-1.471.810.65  (0.419)-1.331.860.00  (1.000)  BMI x  energy density2.652.201.41  (0.235)---  random effectvarianceSDvarianceSD  participant4.252.06-4.672.18-  residual11.963.46-12.023.05-  Table2b: regression models for visit count  visit count  model 1model 2  fixed effectsestimateSEΧ² (p)estimateSEΧ² (p)  intercept3.120.4424.35  (<.001)3.090.4624.25  (<.001)  BMI-.0530.520.99  (0.319)-0.220.420.41  (0.522)  energy  density-0.520.283.40  (0.065)-0.400.252.55  (0.110)  companion (partner)-0.410.530.58  (0.447)-0.590.531.37  (0.242)  gender-0.810.452.988  (0.084)-0.770.462.97  (0.085)  BMI x  energy density0.550.570.85 | *Thank you for taking into consideration of my suggestion and conducted additional analysis using dichotomous variables.*  *First off, I want to note that the presentation of your regression results were messy. You should preview your response file before submitting. Because the results shown in Tables 1a, 1b, 2a, and 2b are not really in a table format, it took me some time to figure out which number corresponds to what. If my understanding is correct, Model 1 in Tables 1a, 1b, 2a, 2b incorporated interaction terms and thus parallel/comparable to Table 3 in your manuscript.*  *By comparing Model 1 results with Table 3, I understand that using dichotomous variables does not significantly alter the results and thus all the results in Table 3 are retained (i.e., MBI, energy density as continuous variables).*  *However, you may already realize that your Tables 1a, 1b, 2a, 2b results are actually more consistent/comparable with Figure 3. This is because intercepts in your regression measure exactly the FD, VD, FC and VC of your base group, adding (or subtracting if negative) the coefficients of BMI, and ED provides estimates for the other group.*  *Figure 3 are plots based on raw data, while results in Tables 1a, 1b, 2a, 2b control for individual characteristics. Hopefully this explanation make sense to you why I wanted to see regression results based on dichotomous groups.* |
